# Molasses as a source of carbon dioxide for attracting the malaria mosquitoes *Anopheles gambiae* and *Anopheles funestus*

**DOI:** 10.1186/1475-2875-13-160

**Published:** 2014-04-27

**Authors:** Collins K Mweresa, Philemon Omusula, Bruno Otieno, Joop JA van Loon, Willem Takken, Wolfgang R Mukabana

**Affiliations:** 1 International Centre of Insect Physiology and Ecology, P.O. Box 30772–00100, GPO, Nairobi, Kenya; 2 Laboratory of Entomology, Wageningen University, PO Box 8031, 6700 EH Wageningen, The Netherlands; 3 School of Biological Sciences, University of Nairobi, PO Box 30197–00100, GPO, Nairobi, Kenya

**Keywords:** Carbon dioxide, Sugar, Sucrose, Molasses, *Anopheles gambiae*, *Anopheles funestus*, Malaria, Mosquitoes, Attraction, Behaviour

## Abstract

**Background:**

Most odour baits for haematophagous arthropods contain carbon dioxide (CO_2_). The CO_2_ is sourced artificially from the fermentation of refined sugar (sucrose), dry ice, pressurized gas cylinders or propane. These sources of CO_2_ are neither cost-effective nor sustainable for use in remote areas of sub-Saharan Africa. In this study, molasses was evaluated as a potential substrate for producing CO_2_ used as bait for malaria mosquitoes.

**Methods:**

The attraction of laboratory-reared and wild *Anopheles gambiae* complex mosquitoes to CO_2_ generated from yeast-fermentation of molasses was assessed under semi-field and field conditions in western Kenya. In the field, responses of wild *Anopheles funestus* were also assessed. Attraction of the mosquitoes to a synthetic mosquito attractant, Mbita blend (comprising ammonia, L-lactic acid, tetradecanoic acid and 3-methyl-1-butanol) when augmented with CO_2_ generated from yeast fermentation of either molasses or sucrose was also investigated.

**Results:**

In semi-field, the release rate of CO_2_ and proportion of *An. gambiae* mosquitoes attracted increased in tandem with an increase in the quantity of yeast-fermented molasses up to an optimal ratio of molasses and dry yeast. More *An. gambiae* mosquitoes were attracted to a combination of the Mbita blend plus CO_2_ produced from fermenting molasses than the Mbita blend plus CO_2_ from yeast-fermented sucrose. In the field, significantly more female *An. gambiae sensu lato* mosquitoes were attracted to the Mbita blend augmented with CO_2_ produced by fermenting 500 g of molasses compared to 250 g of sucrose or 250 g of molasses. Similarly, significantly more *An. funestus*, *Culex* and other anopheline mosquito species were attracted to the Mbita blend augmented with CO_2_ produced from fermenting molasses than the Mbita blend with CO_2_ produced from sucrose. Augmenting the Mbita blend with CO_2_ produced from molasses was associated with high catches of blood-fed *An. gambiae s.l.* and *An. funestus* mosquitoes.

**Conclusion:**

Molasses is a suitable ingredient for the replacement of sucrose as a substrate for the production of CO_2_ for sampling of African malaria vectors and other mosquito species. The finding of blood-fed malaria vectors in traps baited with the Mbita blend and CO_2_ derived from molasses provides a unique opportunity for the study of host-vector interactions.

## Background

Female mosquitoes rely mainly on odour cues to locate vertebrate hosts from which they obtain blood meals necessary for egg development [1]. Carbon dioxide (CO_2_) is the cue that is responsible for activating and guiding mosquitoes towards vertebrate hosts [2-4]. For this reason CO_2_ is commonly added to traps in order to increase mosquito catches during surveillance and/or sampling exercises [5-7]. Previous studies have shown that utilization of CO_2_ to increase trap catches is dependent on the genetic variability among species, the release rate of the gas and the structure of the host-odour plume [3,8-10]. Several studies have demonstrated that CO_2_ enhances trap catches of the anthropophilic and anthropophagic malaria vector *Anopheles gambiae sensu stricto* when released together with human-related odorants [10-12].

Laboratory findings indicated that higher catches of *An. gambiae* mosquitoes were recorded in traps baited with CO_2_ combined with skin emanations or ammonia plus L-lactic acid than CO_2_ alone [9]. Under semi-field conditions, responses of *An. gambiae* to traps baited with foot odour were greatly increased by adding CO_2_[13]. In The Gambia, addition of CO_2_ to synthetic odours substantially increased the catches of females of all mosquito species collected in MM-X traps [12,14]. In addition, traps baited with CO_2_ recorded significantly higher numbers of female *Anopheles gambiae sensu lato* (*s.l.*) and *Anopheles funestus* than those without [3,15]. In many recent studies CO_2_ has been incorporated in synthetic odour blends for sampling malaria vectors [16-18].

Artificial sources of CO_2_ such as fermentation of refined sugar (‘sugar/sucrose’), dry ice and CO_2_ released from pressurized gas cylinders or from propane-powered traps are commonly used in mosquito traps [13,15,19-21]. However, utilization of CO_2_ from these sources is neither cost-effective nor sustainable for mass deployment of odour-baited devices in remote areas of the tropics. For instance, combustion of propane depends on costly gas tanks that are not widely available in rural sub-Saharan Africa where the greatest burden of malaria occurs. In the tropics, dry ice sublimes faster than in temperate areas and therefore, it has to be replaced more frequently. Where mass trapping is required, the use of industrially produced CO_2_ stored in pressurized cylinders will be prohibitively expensive. To overcome these limitations, CO_2_ produced by fermentation of sugar is currently used to supplement synthetic odour-baited MM-X traps for sampling the African malaria vector *An. gambiae* and other mosquito vectors [11]. Nevertheless, increased cost of living and escalating prices of refined cane sugar in sub-Saharan Africa call for production of CO_2_ from cheaper and locally available raw materials.

In this study the possibility of producing CO_2_ by fermentation of sugar cane molasses (i.e., a by-product formed after crystallization of refined white sugar from the raw juice of crushed sugar cane) instead of refined sugar was explored. The objectives of this study were to: (a) determine average release rates of CO_2_ produced by fermentation of different quantities of molasses and dry yeast; (b) evaluate the effect of release rates of CO_2_ on behavioural responses of *An. gambiae;* (c) evaluate the effect of CO_2_ produced from molasses on attractiveness of *An. gambiae* to a previously charatecterized synthetic odour blend; (d) assess the effect of CO_2_ released from refined sugar and molasses on attractiveness of a synthetic odour blend to *An. gambiae*; and, (e) evaluate the effect of CO_2_ released from refined sugar and molasses on attractiveness of a synthetic odour blend to outdoor-biting malaria and other mosquitoes.

## Methods

Semi-field experiments were conducted between April and September 2011 at the Thomas Odhiambo Campus (TOC) of the International Centre of Insect Physiology and Ecology (*icipe*) located near Mbita Point Township in western Kenya. The experiments were aimed at evaluating molasses as a potential substrate for producing CO_2_ for use as a bait for malaria mosquitoes.

### Mosquitoes

Laboratory-reared *An. gambiae* (Mbita strain) mosquitoes were used. Mosquito larvae were raised within a screen-walled greenhouse under ambient climatic conditions. Adult mosquitoes were placed in a holding room under ambient conditions with a photo:scotophase of 12:12 h. Female adult mosquitoes were fed three times a week on blood by direct imbibition from a human arm for 10 min for egg development. Eggs were laid on moist filter paper and dispensed into plastic trays containing filtered water from Lake Victoria. Newly hatched larvae were transferred into plastic basins and fed on Tetramin® baby fish food (Melle, Germany). The larval food was provided three times per day. Pupae were collected daily, placed in clean cups containing filtered water from Lake Victoria and enclosed in mosquito cages. Emerging adult mosquitoes were kept inside cages (30 × 30 × 30 cm) covered by mosquito netting and maintained on 6% glucose solution delivered on Whatman filter paper wicks. Water was provided on cotton towels placed on top of the mesh-covered cages. A total of 200 females aged three to five days old without prior access to a blood meal were randomly collected from holding cages, placed in a release cup covered with mosquito netting, starved for eight hours and released at the centre of a screen-walled greenhouse at the onset of each experiment (20:00–06:30) [22]. During starvation, mosquitoes were only provided with water on a moistened towel.

### Field study site

Field studies were carried out in November 2011 at Kigoche village, situated near Ahero town, in the Kano plains of Kisumu County, western Kenya. Kigoche village is located 00°34′S, 34°65′ E and 1,158 m above sea level, along the northern boundary of the Ahero rice irrigation scheme [18,23], approximately 110 km east of the *icipe,* TOC campus. The area receives between 1,000 and 1,800 mm of rainfall annually with annual temperature and relative humidity (RH) ranges of 17-32°C and 44-80%, respectively. The long rainy season occurs between March and August while short rains are common in October to November. Irrigated rice farming is the main economic activity. Supplementary traditional farming of maize, millet, bananas, sweet potatoes, beans, cassava, sorghum, and rearing of indigenous cattle, goats, sheep, and poultry is also practiced. During the night cattle, sheep and goats are tethered outdoors adjacent to houses occupied by dwellers. Most houses consist of mud walls, corrugated iron-sheet roofs and have either one or two rooms. The houses typically have open eaves spaces but no ceiling. Malaria is mainly transmitted by *An. gambiae, Anopheles arabiensis* and *An. funestus* mosquitoes [18,23,24]. These vectors breed predominantly in rice paddies and in both shaded and unshaded irrigation water channels.

### Preparation and dispensing of synthetic mosquito odour blend

Carbon dioxide was produced from a mixture of sucrose obtained locally from refined cane sugar (‘sugar’) or molasses from sugar cane (‘molasses’) (Mumias Sugar Company Ltd, Kenya), instant dry yeast (‘yeast’) (Angel® Company, China) and water. The sugar content, total dissolved solids and purity of molasses used were determined at the Kenya Sugar Research Foundation (KESREF) laboratory in Kibos, western Kenya. CO_2_ from sugar was produced by mixing 250 g sugar, 17.5 g yeast and 2 L water [11,18]. Molasses-produced CO_2_ was obtained by mixing 2 L water with (a) 125 g molasses and either 8.75 g or 17.5 g yeast, (b) 250 g molasses and 17.5 g or 35 g yeast, and (c), 500 g molasses plus 17.5 g or 35 g yeast. Although tap water was used during semi-field experiments, all field bioassays were conducted using locally available clean water from Kigoche village. The ingredients used to produce CO_2_ were mixed by shaking for 30 sec. The process was carried out in 5 L plastic containers under ambient climatic conditions.

A strip of laboratory Parafilm ‘M’ (Pechiney Plastic Packaging, Chicago, IL 60631, USA) was tied round the connection points along the CO_2_ delivery system to prevent leakage. Non-perfumed pure petroleum jelly (Vaseline™, Unilever Kenya Ltd) was also applied to prevent leakage. There was no more shaking of ingredients in the container upon commencement of CO_2_ emission. Released CO_2_ was delivered through a 60-cm long silicon tubing (0.5 cm diameter) into individual MM-X traps (American Biophysics, North Kingstown, RI, USA) and dispensed singly or in combination with a mosquito attractant referred to as the Mbita blend. In this study, the blend contained ammonia (2.5%), L-lactic acid (85%), tetradecanoic acid (0.00025%) and 3-methyl-1-butanol (0.000001%) each impregnated on a separate nylon strip [25]. The four impregnated nylon strips were hooked together on a wire ring and hung inside the plume tube of a MM-X trap supplied with CO_2_ from either molasses or sugar. The lower end of the plume tube was suspended 15 cm above ground level [26]. Although the treated nylon strips for individual sets of experiments were re-used throughout without replacement or replenishment, fresh CO_2_ was prepared for each experimental replicate [25].

### General procedures

Semi-field experiments were started at 20.00 when it was completely dark. This helped to exclude the possibility of experimental mosquitoes released from the centre of the screen-house from responding to a unidirectional source of light occasioned by sunset. The start of experiments in darkness was not necessary under field conditions (i.e.18:30–06:30) because the wild mosquitoes lured to trapping devices originated randomly from sources located in different directions.

All MM-X traps were operated on 12 V. Vaseline pure petroleum jelly was also applied on suspension wire bars and electrical cables to prevent ants from preying on mosquitoes caught in the MM-X traps. Baited traps and an unbaited MM-X trap were randomly assigned and alternated daily between trap positions to eliminate confounding effects associated with site. A data logger (Tinytag® Ultra, model TGU-1500, INTAB Benelux, the Netherlands) was used to record ambient temperature and RH at an interval of 30 min. To terminate individual experiments, a plug was inserted into the outer tube of the MM-X trap, the CO_2_ supply cut off, and power switched off (semi-field) or traps disconnected from batteries (field study). Latex gloves were worn during preparation of refined sugar/molasses-yeast mixtures, nylon strips, application of attractants on nylon strips and baiting of traps to avoid contamination with human volatiles or other odorant compounds. Traps containing mosquitoes were placed in a refrigerator at -4°C for 30 min. Immobilized mosquitoes were collected from each trap, counted and recorded. Thereafter, traps were cleaned using 70% methanol (to remove residual odours) between experiments. A manual, hand held aspirator was used to collect untrapped, free-flying mosquitoes from the screen-walled greenhouse and killed. The sand-filled floor of the greenhouse was moistened daily to enhance survival of mosquitoes.

### Determination of average release rates of CO_2_ from molasses

The relative amounts of molasses and yeast required to produce an optimal quantity of CO_2_ necessary to elicit significantly high trap catches of *An. gambiae* were evaluated. The CO_2_ produced by fermentation of 250 g sugar mixed with 17.5 g yeast and 2 L water was used as a reference treatment [11]. On this basis, the quantities of molasses and yeast were halved, doubled or kept similar to those in the reference treatment. Two L of water were used in each treatment. The average volume and duration of CO_2_ produced by mixing 2 L water with (a) 125 g molasses plus 8.75 g yeast, (b) 125 g molasses plus 17.5 g yeast, (c) 250 g molasses plus 17.5 g yeast, (d) 250 g molasses plus 35 g yeast, (e) 500 g molasses plus 17.5 g yeast, and (f) 500 g molasses plus 35 g yeast was determined at ambient climatic conditions within a screen-walled greenhouse. The time interval between mixing of ingredients and release of the first bubble of CO_2_ was also recorded.

A 60-cm long silicon tube (0.5 cm diameter) was used to lead CO_2_ into a calibrated beaker held upside down in a plastic basin containing 10 L water. The quantity of CO_2_ released was estimated by measuring and recording volumes of displaced water at intervals of 20 min until the end of each experiment [11]. Individual experiments were replicated four times. A digital stopwatch was used to record the time taken prior to and during CO_2_ production. The presence of CO_2_ in the volatile organic compounds (VOCs) produced by fermenting molasses with yeast was confirmed by the formation of a white precipitate of calcium carbonate when the VOCs were passed through a calcium hydroxide solution. Selection of a suitable combination of molasses and yeast for substituting refined sugar as an alternative source of CO_2_ was based on the length of the release period, volume of CO_2_ produced, release rate, relative attractiveness to *An. gambiae*, bulk and cost-effectiveness.

### Effect of release rates of CO_2_ from molasses on catches of *Anopheles gambiae*

The attraction of *An. gambiae* mosquitoes to traps containing different amounts of CO_2_ produced by varying the quantities of molasses and yeast used as raw materials was compared to those attracted to CO_2_ derived from sugar. These evaluations were done under semi-field conditions. Individual experiments were achieved through dual-choice assays repeated over four nights. The traps assigned for each treatment were placed 10 m apart in diagonal positions within a screen-walled greenhouse. The total number of mosquitoes caught in two unbaited MM-X traps was also recorded to determine the symmetry of the experimental set up [11,27]. The MM-X traps were baited with CO_2_ emitted by a mixture of 2 L water, yeast, and refined sugar or molasses after an incubation period of 30 min (for 250 g sugar and 250 g or 500 g molasses) or 40 min (for 125 g molasses) prior to the onset of each experiment.

### Responses of *Anopheles gambiae* to a synthetic odour blend containing CO_2_ derived from molasses

Semi-field experiments were conducted to ascertain whether attraction of *An. gambiae* mosquitoes to CO_2_ released separately by two promising combinations of molasses and yeast was enhanced by the addition of the Mbita blend. These combinations were 250 g molasses plus 17.5 g yeast, and 500 g molasses plus 17.5 g yeast. Therefore, two sets of dual-choice experiments were designed to investigate the responses of *An. gambiae* to CO_2_ released from (a) 250 g molasses, 17.5 g yeast and 2 L water alone, *versus* 250 g molasses, 17.5 g yeast and 2 L water, in conjunction with the Mbita blend, and (b) 500 g molasses, 17.5 g yeast and 2 L water alone, *versus* 500 g molasses, 17.5 g yeast and 2 L water, presented in conjunction with the Mbita blend.

### Effect of CO_2_ released from sugar and molasses on the attractiveness of a synthetic odour blend to *Anopheles gambiae*

Follow-up experiments were conducted to evaluate responses of *An. gambiae* to the Mbita blend augmented singly with CO_2_ produced by fermentation of 250 g sugar, 250 g molasses or 500 g molasses (each mixed with 17.5 g yeast and 2 L water). This was achieved through a complete 4 × 4 Latin square experimental design replicated for 16 nights. The treatments included a MM-X trap (i) without odour (control), (ii) Mbita blend plus CO_2_ released from 250 g sugar, (iii) Mbita blend plus CO_2_ emitted from 250 g molasses, and (iv) Mbita blend plus CO_2_ released from 500 g molasses. These semi-field studies were subsequently validated under field conditions.

### Responses of wild female malaria vectors

This study was designed to compare the effect of CO_2_ produced by fermentation of 250 g sugar, 250 g molasses or 500 g molasses (each mixed with 17.5 g yeast and 2 L water) on the attractiveness of the Mbita blend to outdoor-biting malaria and other mosquitoes. The treatments included a MM-X trap (i) without odour, (ii) with Mbita blend containing CO_2_ emitted from 250 g sugar, (iii) Mbita blend containing CO_2_ released from 250 g molasses, and (iv) Mbita blend containing CO_2_ derived from 500 g molasses. A randomized 4 × 4 Latin square experimental design replicated over 20 nights was adopted.

**Figure 1:**
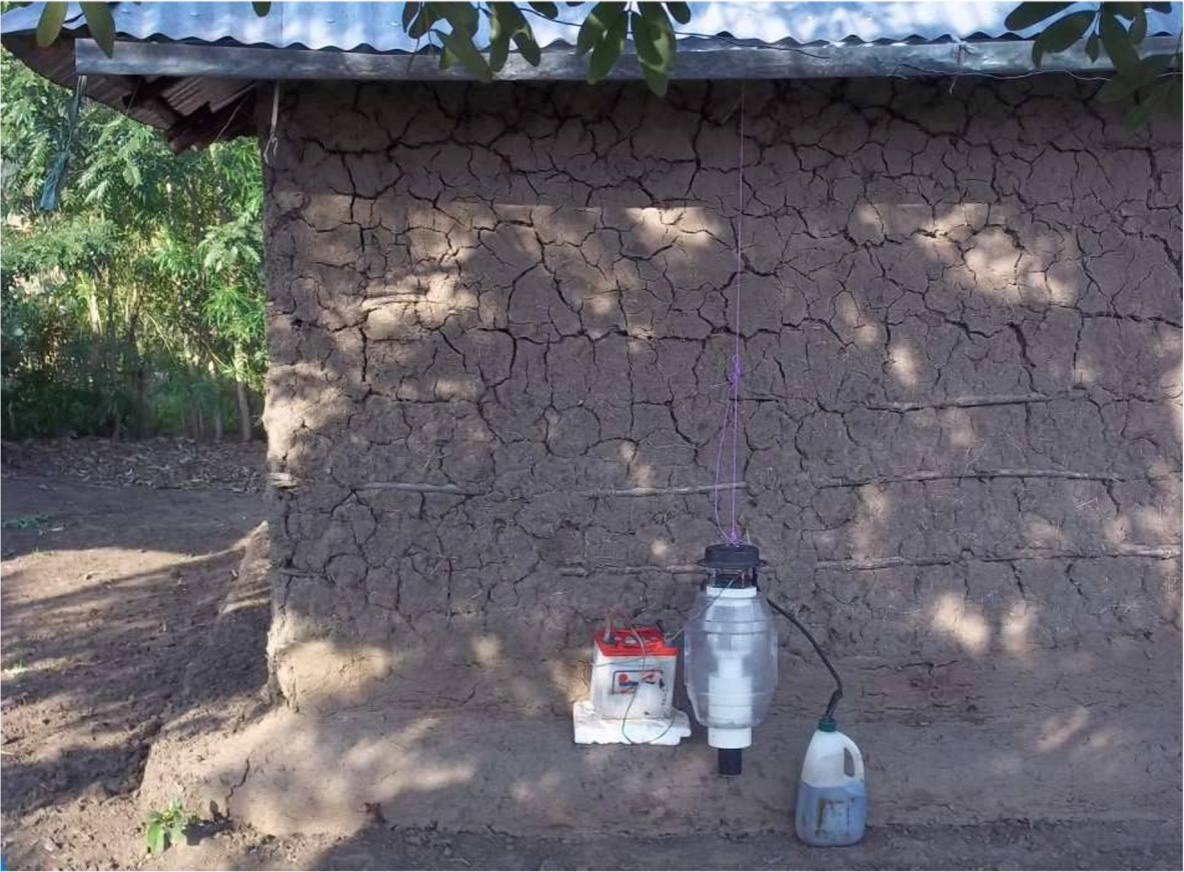
**A picture of an outdoor trapping system baited with the Mbita odour blend augmented with carbon dioxide produced by fermentation of 250 g molasses using 17.5 g dry yeast mixed with 2 L water.** Carbon dioxide was produced in a plastic container and delivered into the MM-X trap through silicon tubing.

Individual treatments were assigned to particular MM-X traps suspended outside the bedroom under the eaves of village houses (Figure 1). Each house was used and occupied routinely by two to five dwellers. The dwellers slept under untreated bed nets during experimental nights [12]. The four village houses used in the field experiment were spaced at least 40 m away from one another. This excluded the potential interaction of treatments placed in any two adjacent houses. Treatments in the 4 × 4 Latin square experimental design were allocated with respect to day and position. Thus, individual treatments were rotated between houses every day. One experimental round lasted four days and this equates to 16 days for a fully replicated 4 × 4 Latin square experiment.

At the end of each experimental night, all traps were transported to a field laboratory located at the Ahero Multipurpose Development Training Institute (AMDTI) and placed in a freezer for 30 min. The frozen adult mosquitoes were emptied into labeled Petri dishes, identified morphologically [28] counted, and recorded according to (i) sex as male or female *An. gambiae s.l.*, *An. funestus*, *Culex*, *Mansonia* spp. and other anopheline mosquitoes (all collected *Anopheles* spp. except *An. gambiae s.l.* and *An. funestus*) and (ii) external abdominal appearance as unfed, blood-fed (involved fully and partially blood-fed), or gravid females of *An. funestus*, and *An. gambiae s.l.*[29]. All female *An. gambiae s.l.* and *An. funestus* were preserved in 2 mL Eppendorf tubes containing silica gel crystals.

### Ethical approval

Scientific and ethical approval of the present study was granted by the Kenya Medical Research Institute (KEMRI/RES/7/3/1). Inclusion consent of houses into the study was obtained from household heads and the local administration.

### Data analysis

Differences in the release rates of CO_2_ produced from 250 g sugar, 125 g, 250 g and 500 g molasses were determined by using a General Linear Model (GLM), univariate analysis of variance. The Tukey test was used for pairwise comparison of release rates of CO_2_ from sugar *versus* 125 g, 250 g and 500 g molasses. Individual dual-choice bioassays were analyzed using Chi-square tests. The Chi-square test determined whether the distribution of total number of mosquitoes caught in both MM-X traps differed from a 1:1 distribution [11]. Trap counts of mosquitoes collected from experiments conducted through a 4× 4 Latin square design were analysed using a GLM assuming a Poisson distribution and logarithmic link function [22]. The effects of treatment, trap position or house on mosquito catches were tested and fitted as parameters in the model. The significance of trap or house on mosquito catches was fitted with treatment in the model to test for interaction. Effects were considered significant at P <0.05. All analyses were performed using IBM SPSS statistical software, version 16.

## Results

### Determination of average release rates of CO_2_ from molasses

**Figure 2:**
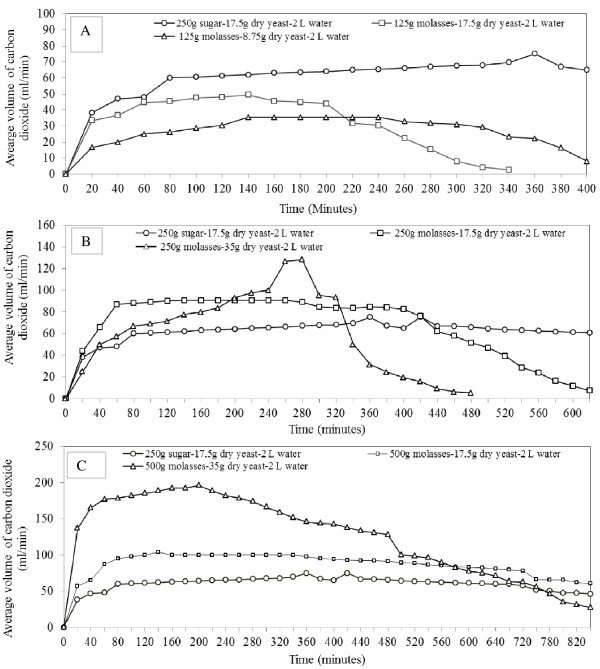
Average release rate (ml/min) of carbon dioxide produced by 2 L water mixed with different quantities of yeast and 125 g (panel A), 250 g (panel B), or 500 g (panel C) molasses compared to a combination of 250 g refined sugar, 17.5 g dry yeast and 2 L water (reference treatment).

An average temperature of 23.2 ± 1.3°C and 77.0 ± 2.6% RH were recorded during semi-field experiments (April-September 2011). The molasses used in all experiments was 44.7% pure, and contained 34.2% sugar and 76.4% dissolved solids. The release rates of CO_2_ were dependent on the quantity of molasses used (P <0.001). Fermentation of 125 g, 250 g and 500 g molasses emitted CO_2_ within a time range of 310 to 440 min, 490 to 645 min, and >840 min, respectively (Figure 2). An increase in the quantity of molasses enhanced release rates and duration of CO_2_ production. The average post-mixing time range for emission of a first bubble of CO_2_ from 125 g, and 250 g to 500 g molasses was 29.00-37.50 min, 20.00-29.50 min and 18.35-26.09 min, respectively.

Although release rates of CO_2_ from 125 g molasses mixed with 8.75 g (36.5 ± 3.3 ml/min) or 17.5 g (30.3 ± 1.6 ml/min) yeast were not different (P = 0.12), the two release rates were significantly lower compared to 250 g sugar + 17.5 g yeast (63.23 ± 2.82 ml/min) (P <0.001) (Figure 2A). There was a greater increase in the release rate of CO_2_ produced by a combination of 250 g molasses + 17.5 g yeast (80.63 ± 2.82 ml/min) compared to 250 g sugar or 250 g molasses + 35 g yeast (63.9 ± 6.6 ml/min) (P = 0.010 for both) (Figure 2B). By contrast, release rates of CO_2_ obtained from 250 g sugar, and 250 g molasses + 35 g yeast were not different (P = 0.10). The release rate of CO_2_ was greatly increased by mixing 500 g molasses with either 17.5 g (87.79 ± 2.14 ml/min) or 35 g (127.4 ± 8.9 ml/min) yeast compared to 250 g sugar (P <0.001 for both) (Figure 2C).

### Effect of release rates of CO_2_ from molasses on catches of *Anopheles gambiae*

Trap positions had no influence on mosquito catches within the screen-walled greenhouse (P = 0.11). Both traps without odour caught 15.5% (n = 124) of the released mosquitoes implying that all bioassays were symmetrical. There was a significant increase in the proportions of mosquitoes attracted to CO_2_ released from 250 g sugar + 17.5 g yeast than to a mixture of 125 g molasses + 8.75 g yeast (P <0.001). However, the attractiveness of CO_2_ produced by fermentation of 250 g sugar + 17.5 g yeast, and 125 g molasses + 17.5 g yeast to mosquitoes was not different (P = 0.51). A significantly higher proportion of mosquitoes responded to CO_2_ released from 250 g molasses + 17.5 g yeast compared to 250 g sugar + 17.5 g yeast (P < 0.001). By contrast, mosquitoes responded equally to CO_2_ derived from 250 g sugar + 17.5 g yeast and 250 g molasses + 35 g yeast (P = 0.07). Nonetheless, there was a higher response of mosquitoes to CO_2_ emitted from a mixture of 500 g molasses + 17.5 g yeast than to CO_2_ released from 250 g sugar + 17.5 g yeast (P = 0.001). Although the release rate of CO_2_ from 500 g molasses + 35 g yeast was the highest, this combination attracted similar proportions of mosquitoes compared to 250 g sugar + 17.5 g yeast (P = 0.11). These experiments (Table 1) provided the baseline information used to select potential combinations of molasses and yeast for replacement of the currently used 250 g sugar as a source of CO_2_ bait for sampling malaria vectors.

**Table 1:** **Total and mean (±SE) number of *Anopheles gambiae *** **attracted by carbon dioxide produced in a dual-choice assay in a screen house between molasses treatments (test combinations of molasses and dry yeast dissolved in 2 L water) and a reference treatment (250 g refined sugar, 17.5 g dry yeast and 2 L water)**

**Molasses treatments**	**N**	**n**	**Mean ± SE number of mosquitoes attracted**		**P-value**
			**Molasses treatment**	**Reference treatment**	
125 g molasses - 17.5 g yeast - 2 L water	4	507	61.5 ± 4.0	65.3 ± 4.0	0.51
125 g molasses - 8.75 g yeast - 2 L water	4	417	35.8 ± 3.0	68.5 ± 4.1	0.001
250 g molasses - 17.5 g yeast - 2 L water	4	460	70 ± 4.2	45.0 ± 3.4	0.001
250 g molasses - 35 g yeast - 2 L water	4	371	50.8 ± 3.6	42.0 ± 3.2	0.07
500 g molasses - 17.5 g yeast - 2 L water	4	545	79.3 ± 4.5	57.0 ± 3.8	0.001
500 g molasses - 35 g yeast - 2 L water	4	440	50.8 ± 3.6	59.3 ± 3.9	0.11

N is the number of experimental nights, n is the total number of mosquitoes caught whereas SE is the standard error of the mean catch per night. A total of 200 female *An. gambiae* were released per night. Pairwise comparisons in the same row of mean catches differ significantly at P <0.05 (Chi-square test).

### Responses of *Anopheles gambiae* to a synthetic odour blend containing CO_2_ derived from molasses

**Figure 3:**
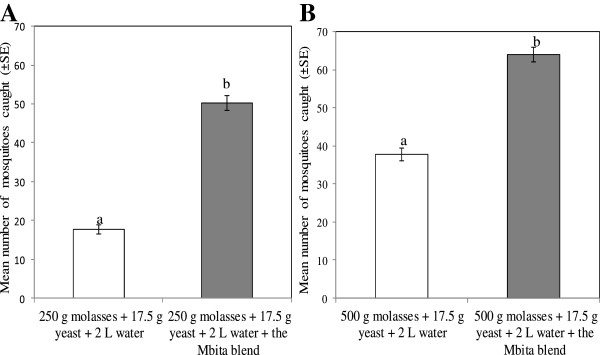
**Effect of adding the Mbita odour blend to carbon dioxide produced by fermentation of 250 g (panel A) and 500 g (panel B) molasses on the mean number (±SE) of *Anopheles gambiae *collected for four nights.** A total of 200 female *An. gambiae* were released per night within a screen-walled greenhouse. Bars with different letters in the same graph differ significantly (P <0.05). Error bars represent the standard error of the mean number of mosquitoes collected.

Preceding results indicated that fermentation of 250 g or 500 g molasses (each mixed with 17.5 g yeast and 2 L water) were promising alternatives for replacing the currently used combination of sugar (250 g), yeast (17.5 g) and water (2 L) to produce CO_2_. The responses of mosquitoes to CO_2_ produced from either 250 g or 500 g molasses was significantly increased when released in conjunction with the Mbita odour blend compared to CO_2_ alone from either quantity (P < 0.001 for both). Of the 272 trapped mosquitoes, 26.1% were attracted to CO_2_ released from 250 g molasses and 73.9% to the Mbita blend augmented with CO_2_ derived from 250 g molasses (Figure 3A). In a similar bioassay, of the 407 mosquitoes collected, 37.1% were attracted to CO_2_ emitted from 500 g molasses, and 62.9% to the Mbita blend supplemented with CO_2_ released from 500 g molasses (Figure 3B).

### Effect of CO_2_ released from sugar and molasses on the attractiveness of a synthetic odour blend to *Anopheles gambiae*

Out of the 3,200 *An. gambiae* mosquitoes released, 1,801 (i e, 56.3%) were trapped during 16 experimental nights. The mosquitoes were collected in traps without odour (1.4%) or traps containing the Mbita blend augmented with CO_2_ released from 250 g sugar (25%), 250 g molasses (33.9%) or 500 g molasses (38.9%) (Table 2). Addition of CO_2_ from 250 g molasses to the Mbita blend attracted more mosquitoes than when the Mbita blend was combined with CO_2_ from 250 g sugar (P <0.001). Similarly, the attractiveness of the Mbita blend was significantly increased by adding CO_2_ produced by fermentation of 500 g molasses than from 250 g sugar (P <0.001). The attractiveness of the Mbita blend was greatly enhanced by the addition of CO_2_ released from 500 g fermenting molasses than 250 g molasses (P <0.014).

**Table 2:** **Total and mean (±SE) number of *Anopheles gambiae *** **caught in a screen house using a MM-X traps without odour, baited with the Mbita odour blend (MB) augmented with carbon dioxide produced by fermentation of either 250 g refined sugar, 250 g or 500 g molasses by using dry yeast**

**Treatment**	**N**	**Mosquitoes caught**	
		**n**	**Mean (±SE)**
No odour	16	25	1.7 ± 0.3^a^
250 g refined sugar - 17.5 g yeast - 2 L water and Mbita blend	16	465	28.8 ± 1.3^b^
250 g molasses - 17.5 g yeast - 2 L water and Mbita blend	16	611	37.8 ± 1.5^c^
500 g molasses - 17.5 g yeast - 2 L water and Mbita blend	16	700	43.4 ± 1.6^d^

N is the number of experimental nights, n is total number of mosquitoes caught whereas SE is the standard error of the mean catch per night. A total of 200 female *An. gambiae* were released per night. Mean catches in the same column with different superscript letters differ significantly at P <0.05 (Generalized Linear Models).

### Responses of wild female malaria vectors

**Figure 4:**
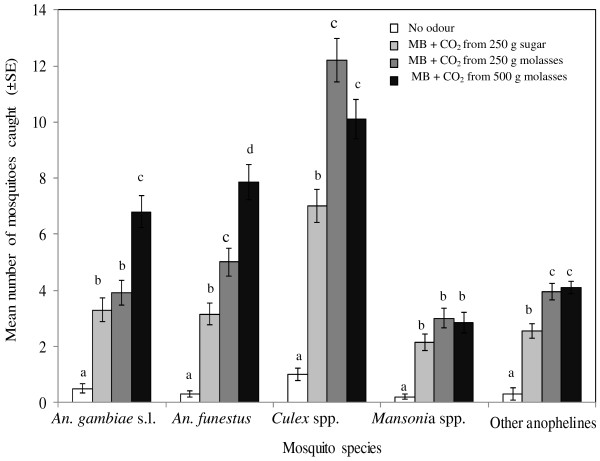
**Mean number (±SE) of female mosquitoes caught overnight outdoors in MM-X trap without odour, baited with the Mbita blend (MB) augmented with carbon dioxide produced by fermentation of either 250 g refined sugar or 250 g and 500 g molasses.** Mean values within the same mosquito type with different letters are significantly (P < 0.05) different.

The 20 nights of field experiments (November 2011) were characterized by a mean temperature of 23.5 ± 2.2°C, 71.9 ± 1.9% RH, wind speed of 3.2 ± 0.09 km/h, and total rainfall of 263.4 mm. A total of 1,807 mosquitoes were caught outdoors. Of this number, 11.2% (n = 203) were males and 88.7% (n = 1,604) were females. Both treatment and house effects played an important role in influencing trap collections of all female mosquitoes (P <0.001 for both). The 1,604 female mosquitoes were collected in traps without odour (2.9%), baited with the Mbita blend supplemented with CO_2_ released by fermenting 250 g sugar (22.6%), fermenting 250 g molasses (35.0%) and fermenting 500 g molasses (39.5%) (Figure 4). The female mosquitoes comprised *An. gambiae s.l.* (18.1%), *An. funestus* (20.2%), *Culex* spp. (37.8%), *Mansonia* spp. (10.2%) and other anophelines (13.6%).

Trap collections of female *An. gambiae s.l.* and *An. funestus* were influenced by the carbohydrate source of CO_2_ presented with the Mbita blend (P <0.001, for both). Addition of CO_2_ released from 500 g molasses to the Mbita blend attracted the highest number of *An. gambiae s.l.* compared to either CO_2_ derived from 250 g molasses (P <0.001) or from 250 g sugar (P <0.001). However, addition of CO_2_ released from 250 g molasses or 250 g sugar on the Mbita blend caused no significant difference on trap collections of *An. gambiae s.l.* (P = 0.32).

There was a great increase in the responses of *An. funestus* to the Mbita blend supplemented with CO_2_ released from 500 g or 250 g molasses than to 250 g sugar (P <0.001 and P <0.007, respectively). Nonetheless, the Mbita blend was more attractive to *An. funestus* when augmented with CO_2_ derived from 500 g than 250 g molasses (P <0.01). Combinations of the Mbita blend and CO_2_ released from 250 g or 500 g molasses were equally attractive to *Culex* spp. (P = 0.12) and other anopheline mosquitoes (P = 0.18). However, attractiveness of the Mbita blend supplemented with CO_2_ from 250 g sugar to *Culex* spp. was significantly lower compared to 250 g (P <0.001) or 500 g (P <0.010) molasses. By contrast, trap collections of *Mansonia* spp. were not dependent on the carbohydrate source of CO_2_ (P = 0.42).

**Figure 5:**
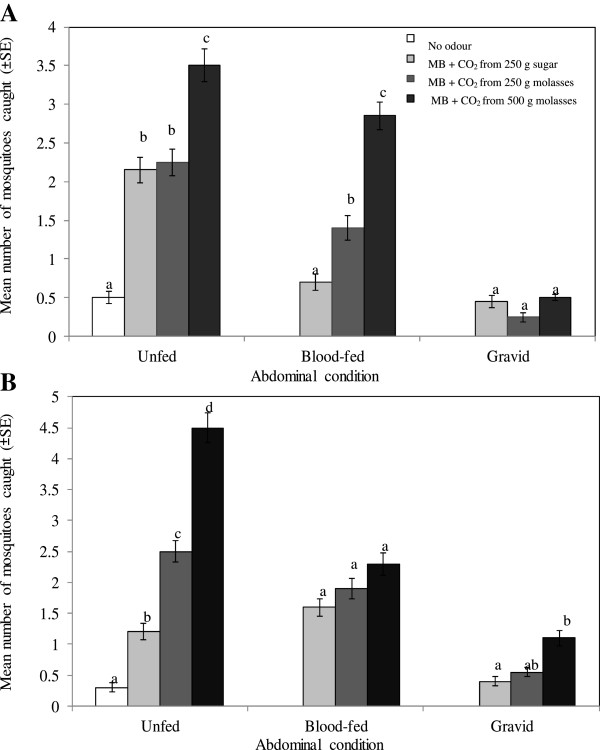
**Mean number (±SE) of outdoor-biting *Anopheles gambiae *s.l. (panel A) and *Anopheles funestus *(panel B) mosquitoes of different abdominal conditions (unfed, blood-fed and gravid).** The mosquitoes were collected overnight in a MM-X trap without odour, baited with the Mbita blend (MB) augmented with carbon dioxide released from 250 g refined sugar, 250 g or 500 g molasses for 20 nights. Treatments are shown in the legend of panel **A**. Mean values within the same mosquito type with different letters are significantly (P <0.05) different.

A combination between the Mbita blend and CO_2_ produced by fermentation of molasses elicited a physiological, stage-dependent behaviour of local malaria vectors. The 290 females of *An. gambiae s.l.* collected were unfed (57.6%), blood-fed (33.8%) or gravid (8.6%) (Figure 5A). The responses of unfed *An. gambiae s.l.* to the Mbita blend were significantly increased by addition of CO_2_ derived from 500 g molasses compared to 250 g molasses (P <0.012) or 250 g sugar (P <0.001). There was no difference in the responses of unfed *An. gambiaes s.l.* to the Mbita blend supplemented with CO_2_ released from either 250 g molasses or 250 g sugar (P = 0.73).

A higher mean number of blood-fed *An. gambiae s.l.* was attracted to the Mbita blend augmented with CO_2_ emitted from 500 g molasses than from 250 g sugar (P <0.001) or from 250 g molasses (P <0.001). Also, the attractiveness of the Mbita blend to blood-fed *An. gambiae s.l.* was significantly enhanced by addition of CO_2_ released from 250 g molasses compared to 250 g sugar (P <0.034). Treatment had no effect on trap catches of gravid *An. gambiae s.l.* (P = 0.34).

The 324 females of *An. funestus* collected were unfed (51.2%), blood-fed (36.1%) or gravid (12.6%) (Figure 5B). The attraction of unfed *An. funestus* to the Mbita blend was greatly enhanced by addition of CO_2_ derived from 250 g or 500 g molasses compared to 250 g sugar (P <0.013 and P <0.001, respectively). However, responses of gravid *An. funestus* to the Mbita blend supplemented with CO_2_ released from 250 g molasses or from 250 g sugar were not different (P = 0.49). By contrast, significantly more gravid *An. funestus* were attracted to the Mbita blend augmented with CO_2_ produced by fermentation of 500 g molasses compared to 250 g sugar (P <0.014). Although baited traps collected high numbers of blood-fed *An. funestus*, the catches were not statistically different (P = 0.14).

## Discussion

The findings of this study indicate that CO_2_, and possibly other volatiles, produced by fermentation of molasses provide a suitable alternative to the CO_2_ obtained from fermentation of the more expensive, refined cane sugar to lure malaria vectors towards traps. The release rate and proportion of *An. gambiae* mosquitoes caught increased consistently as the quantity of yeast-fermented molasses increased up to an optimal ratio of molasses and yeast. The attraction of mosquitoes to traps baited with CO_2_ produced by fermenting molasses was enhanced when presented jointly with the Mbita blend of synthetic odours. More malaria vectors and female *Culex* spp. responded to the Mbita blend supplemented with CO_2_ released from molasses compared to a combination of the Mbita blend and CO_2_ derived from sugar. Total collections of wild mosquitoes comprised of 18.0% *An. gambiae s.l.* and 20.3% *An. funestus* in an unfed, blood-fed or gravid abdominal condition. Significantly more blood-fed *An. gambiae s.l.* were caught in traps baited with CO_2_ derived from fermenting molasses compared to sugar.

The results confirm that variation in the release rates of CO_2_ influenced by ratios of molasses and dry yeast can be sufficiently large to induce differential activation and upwind orientation of mosquitoes towards baited traps [2,4,10]. For example, 125 g molasses resulted in significantly lower release rates of CO_2_ lasting for periods of 320–440 min compared to a higher, stable flow rate and longer release period (over 840 min) demonstrated by the sugar source. Utilization of 250 g molasses mixed with 17.5 g yeast produced CO_2_ for periods of 490–645 min, and this translated into significantly higher mosquito catches compared to those collected with CO_2_ from 250 g sugar with 17.5 g yeast. By contrast, fewer mosquitoes responded to CO_2_ derived from 250 g molasses and 35 g yeast, possibly because of a drastic drop in release rates resulting in an insufficient gas supply to sustain trap collections through the dawn peak biting period. It is also possible that doubling the quantity of yeast enhanced the fermentation rate of molasses, and reduced the release period thereby producing higher concentrations of CO_2_ with an inhibitory effect on host-seeking responses of *An. gambiae*[3,11,30]. Such an inhibitory effect has been reported in other studies [30,31] and it is likely to account for lower mosquito responses to high release rates of CO_2_ from a mixture of 500 g molasses + 35 g yeast in 2 L water. These results indicate that although CO_2_ activates mosquitoes by inducing upwind flight, higher concentrations may reduce orientation to the source at close range [2,32,33]. In fact, some laboratories use high concentrations of pure CO_2_ to anaesthetize mosquitoes.

The release rates of CO_2_ produced by fermentation of 250 g (80.6 ± 2.82 ml/min) or 500 g (87.8 ± 2.14 ml/min) molasses (each mixed with 17.5 g yeast and 2 L water) were not different. However, more mosquitoes responded to CO_2_ produced by fermentation of 500 g than to 250 g molasses, possibly because the higher quantity of molasses provided more substrate for an extended release period of optimal CO_2_ concentrations. In contrast, fermentation of 250 g molasses, despite having a lower sugar content, showed a higher release rate of CO_2_ and was more attractive to *An. gambiae* compared to 250 g refined sugar. Both carbohydrate sources were mixed with the same quantity of yeast and water. This suggests that, besides CO_2,_ yeast-fermented molasses produces additional VOCs that enhance mosquito catches. The VOCs emitted by fermented sugar have been reported [11]. Whereas molasses, unlike sugar, contains a lower sugar content, it has relatively more water, solid matter, nitrogen, and minerals [34].

During the current study, the numbers of *An. gambiae* mosquitoes attracted to CO_2_ obtained from either 250 g or 500 g molasses was greatly increased in the presence of the Mbita blend. This implies that *An. gambiae* mosquitoes locate and orientate themselves towards odour-baited trapping systems by responding to CO_2_ released together with host-specific cues [2,9,22,35]. Thus, the results demonstrate that, although CO_2_ is one of the stimuli to which mosquitoes respond, trap catches are significantly increased in the presence of a plume containing human odour or synthetic odour than on its own [2,9,35]. It has been demonstrated that trap catches of host-seeking *An. gambiae* and *Aedes aegypti* are influenced by the structure of host odour-plumes and that the effect of CO_2_ on mosquito catches is concentration dependent [33]. Moreover, it was also shown that the additive effect of CO_2_ on worn socks is responsible for the attraction of most mosquito species in the genus *Aedes*, *Anopheles*, *Coquillettidia*, *Culex*, *Culiseta*, and *Psorophora*[8].

Similarly, the abundance and diversity of mosquitoes caught outside village houses occupied by dwellers depended on CO_2_ source and dose, hence corresponding to previously reported findings [3]. During the current study, a combination between CO_2_ produced by 500 g fermenting molasses and the Mbita blend led to the highest catches of unfed and blood-fed females of *An. gambiae s.l..* However, the numbers of female *An. gambiae s.l.* collected in a trap baited with the Mbita blend augmented with CO_2_ from 250 g molasses or 250 g sugar were similar, indicating that both CO_2_ sources were equally attractive. Nonetheless, CO_2_ produced by 250 g fermenting molasses attracted similar numbers of female *Culex* spp. and other anopheline mosquitoes as 500 g molasses when presented with the Mbita blend. These observations indicate that CO_2_ produced by 250 g fermenting molasses is a suitable alternative to that produced from 250 g sugar. However, utilization of 500 g molasses would be a convenient alternative for mass-trapping of malaria vectors in situations where malaria-prone areas are endowed with sufficient resources and large scale production of sugar cane. It is then not necessary to increase the quantity of yeast, as it is, in congruence with enzyme kinetics [11,34], shown that 17.5 g yeast added to 2 L water and a sugar source is sufficient for optimal CO_2_ production.

The attraction of significantly higher numbers of blood-fed *An. gambiae s.l.* mosquitoes to the Mbita blend augmented with CO_2_ released from molasses is a very interesting finding. Blood-fed mosquitoes are rarely caught with indoor or outdoor human landing collections [36,37]. The method is also not ethically acceptable unless collectors are given proper prophylaxis against malaria [38]. Instead, more representative samples of unfed, blood-fed and gravid females of *An. gambiae s.l.* as well as males are obtained by collection of resting mosquitoes [39,40]. This accounts for the use of resting boxes, pyrethrum spray catches, clay pots and manual aspiration of resting mosquitoes especially in studies associated with the estimation of the entomological inoculation rate [36,41]. Seemingly, cues derived from a combination of the Mbita blend and CO_2_ accompanied with other VOCs produced from fermenting molasses may have activated a temporal sensitivity of odour receptors thereby inducing host-seeking behaviour of unfed and blood-fed *An. gambiae s.l.* and *An. funestus*[42]. These findings are contrary to the expectations that after a blood meal, blood-feeding responses of female mosquitoes are down regulated for the next 48 to 72 hours until eggs mature and increase sensitivity of odour receptors to oviposition cues [41]. It has also been reported that small-bodied malaria vectors and those that take small-sized blood meals engage more frequently in multiple blood feeding during single gonotrophic cycles to meet their nutritional requirements [43,44]. Such feeding behaviours are more likely to increase human-vector contact and risk of malaria transmission. Whereas there were cows resting adjacent to all houses occupied by dwellers in surroundings where outdoor-biting mosquitoes were collected, utilization of different synthetic odour baits supplemented with CO_2_ derived from refined sugar attracted predominantly unfed *An. gambiae s.l.* and *An. funestus* in the same study site [18]. Thus, there is a high likelihood that the mosquitoes responded differently to the release rates of CO_2_ used to augment the Mbita blend due to genetic variability among species.

From the foregoing it is clear that attraction of blood-fed malaria vectors by synthetic odour blends supplemented with CO_2_ in conjunction with VOCs released from fermentation of molasses requires further investigation. This may provide an important resource for studies that require estimates of malaria transmission risk, as provided by the entomological inoculation rate [45]. The collection of blood-fed mosquitoes can also be used for studies of the infectious reservoir of malaria; as such mosquitoes may have fed on *Plasmodium*-infected hosts. Clearly the additional volatiles produced by fermentation of molasses stimulate blood-fed females to respond to the odour-baited trap, which opens up new potential for malaria-epidemiological studies. In this study no attempts were made to identify other volatile compounds produced by yeast fermentation of molasses. Likewise no efforts were made to remove/separate CO_2_ from the other gaseous products (if any) in order to allow investigations on the potential effect of any other products on eliciting mosquito behavioural responses. Although this research was not aimed at resolving such issues, there is a need for further investigations which should also focus on (a) determination of the relative composition of VOCs emitted by fermenting molasses and their effect on the capture rate of malaria and other mosquito vectors in different physiological conditions, (b) identification of blood meal source, and (c) how this technology may be used for estimation of entomological inoculation rate.

Whereas semi-field bioassays were conducted using *An. gambiae s.s.*, collection of mosquitoes indoors in the same village confirmed that *An. gambiae s.l.* was represented by 96.7% *An. arabiensis* and 3.3% *An. gambiae s.s.*[18]. The study confirms recent findings [11,12] in that, it is possible to intercept and reduce the number of malaria mosquitoes entering or leaving houses by deploying outdoor traps baited with a combination of CO_2_ derived from molasses and synthetic blends that mimic human odour. Deployment of this innovative technology is extremely important against outdoor-biting vectors responsible for maintaining residual transmission of malaria because intensive use of insecticide-treated nets and indoor residual spraying continues to reduce indoor transmission [46-48]. Because baiting of traps with CO_2_ affects host-seeking behaviour of different species of malaria vectors, further reduction of house entry of mosquitoes can be achieved by incorporation of complementary measures such as house screening [49] or application of novel repellents in a push-pull strategy [50].

## Conclusion

Yeast-fermented molasses is an effective alternative source of CO_2_ for odour-baited trapping systems of mosquitoes as it is equally or more attractive than CO_2_ from fermenting sugar. This study presents an alternative use of molasses and a local solution for the development of cheaper and more sustainable lures for sampling and control of *An.* gambiae *s.l., An. funestus,* and other human-biting mosquitoes in rural areas. This research did not expect to find blood-fed mosquitoes inside traps baited with a synthetic odour blend supplemented with a combination of CO_2_ and VOCs released from fermenting molasses as female mosquitoes are not attracted to their vertebrate hosts after blood feeding. Therefore, a need for further investigations on this finding is necessary.

## Competing interests

The authors declare that they have no competing interests.

## Authors’ contributions

CKM, WT and WRM designed the study; CKM, PO and BO conducted the research; WRM and CKM analysed the data; CKM, JJAvL, WT, and WRM wrote the paper. All authors read and approved the final manuscript.
